# Manipulation of Pattern of Cell Differentiation in a *hetR* Mutant of *Anabaena* sp. PCC 7120 by Overexpressing *hetZ* Alone or with *hetP*

**DOI:** 10.3390/life8040060

**Published:** 2018-11-30

**Authors:** He Zhang, Xudong Xu

**Affiliations:** The State Key Laboratory of Freshwater Ecology and Biotechnology, Institute of Hydrobiology, Chinese Academy of Sciences, Wuhan 430072, China; zhanghe1983004@sina.com

**Keywords:** HetR, HetZ, HetP, heterocyst formation, patterning

## Abstract

In the filamentous cyanobacterium, *Anabaena* sp. PCC 7120, single heterocysts differentiate at semi-regular intervals in response to nitrogen stepdown. HetR is a principal regulator of heterocyst differentiation, and *hetP* and *hetZ* are two genes that are regulated directly by HetR. In a *hetR* mutant generated from the IHB (Institute of Hydrobiology) substrain of PCC 7120, heterocyst formation can be restored by moderate expression of *hetZ* and *hetP.* The resulting heterocysts are located at terminal positions. We used a tandem promoter, P*_rbcL_*P*_petE_*_,_ to express *hetZ* and *hetP* strongly in the *hetR* mutant. Co-expression of *hetZ* and *hetP* enabled the *hetR* mutant to form multiple contiguous heterocysts at both terminal and intercalary positions. Expression of *hetZ*, alone resulted in terminally located heterocysts, whereas expression of *hetP*, alone produced enlarged cells in strings. In the absence of HetR, formation of heterocysts was insensitive to the peptide inhibitor, RGSGR.

## 1. Introduction

Cyanobacteria are the most ancient, oxygenic, photosynthetic organisms on earth [1,2]. Some filamentous cyanobacteria produce specialized cells called heterocysts to fix N_2_ under aerobic conditions [3,4]. Heterocyst pattern formation represents one of the earliest multicellular patterning processes in the history of life [5].

*Anabaena* sp. PCC 7120 (*Anabaena* 7120) is a model strain often used for molecular studies of heterocyst differentiation and patterning. In response to nitrogen stepdown, this strain transforms some CO_2_-fixing vegetative cells into single N_2_-fixing heterocysts at semi-regular intervals [3]. Spacing of heterocysts depends largely on an activator-inhibitor patterning system [6,7]. HetR has been proposed to be the master regulator (activator) of heterocyst differentiation [8], and diffusible RGSGR-containing peptides (inhibitor) have been shown to inactivate HetR in vitro [9,10,11] and to induce the degradation of HetR in cells [12]. In addition, gene *patA* [13], which encodes a CheY-like response regulator with a PATAN domain at N-terminus [14], is required for a semi-regular pattern. In a *patA* mutant, heterocysts form almost exclusively at the ends of filaments. Both activation of RGSGR-containing peptide genes in differentiating cells [15,16] and expression of *patA* in vegetative cells [10] are required for normal pattern formation and depend on direct or indirect regulation by HetR [17].

In the wild type of *Anabaena* 7120, HetR accumulates in differentiating cells [18,19] and directly activates the expression of *hetP* [20] and *hetZ* [9]. The latter two genes are required for heterocyst differentiation and mediate the regulation under the control of HetR [21]. In the IHB substrain of *Anabaena* 7120 (*Anabaena* 7120 IHB) [22], co-expression of *hetZ* and *hetP* from P*_ntcA_* restored heterocyst formation at the ends of filaments in the *hetR*::C.CE2 mutant, whereas expression of *hetZ* or *hetP* did not [21]; use of P*_petE_* produced similar results (Zhang and Xu, unpublished). In a different version of *Anabaena* 7120, expression of *hetZ* from P*_petE_* enabled a *hetR* deletion mutant to form multiple contiguous heterocysts (Mch) at intercalary and terminal positions [23]. The two versions of *Anabaena* 7120 appeared to differ partly from each other in the functions of *hetZ* and *hetP*, especially in the extent to which the expression of *hetZ* can bypass the *hetR* mutation.

Because *hetZ* and *hetP* overlap functionally [21], further enhanced expression of *hetZ* may also be able to restore heterocyst formation in the *hetR* mutant. Before the use of P*_ntcA_*_,_ we had used the P*_rbcL_*P*_petE_* tandem promoter to express *hetZ* and (or) *hetP* in the *hetR* mutant of the *Anabaena* 7120 IHB. By expressing *hetZ* or by co-expressing *hetZ* and *hetP* from the tandem promoter, we were able to generate two different patterns in the *hetR* background: single terminal heterocysts and Mch. In addition to *hetR*, *hetZ* and *hetP*, a caspase-hemoglobinase-fold protease gene, called *hetF*, is also required for heterocyst differentiation in *Anabaena* 7120 [24]. In a *hetF*::Tn*5*-1087b mutant, however, P*_rbcL_*P*_petE_*-*hetZ* allowed heterocysts to be formed at both terminal and intercalary positions.

## 2. Materials and Methods

### 2.1. General

*Anabaena* 7120 and derivatives, listed in Table 1, were cultured in BG11 medium [4] in the light, 30 μE m^−2^ s^−1^, on a rotary shaker. Erythromycin (5 μg mL^−1^), neomycin (20 μg mL^−1^) or spectinomycin (10 μg mL^−1^) was added to the medium as appropriate. *Anabaena* filaments were washed 3 times with BG11_0_ (without nitrate) before induction of heterocyst formation in the same medium. Nitrogenase activities were measured as previously described [21].

Alcian blue staining of a heterocyst polysaccharide layer was performed as described by Hebbar and Curtis (2000) [26]. Heterocyst frequencies (>300 cells counted per sample) and frequency distribution of intervals between heterocysts (>1000 cells counted per sample) were analyzed using three cultures in parallel. Data are means ± SD.

### 2.2. Plasmid Construction and Conjugation

Plasmid construction is briefly described here and detailed in Table S1. A PCR fragment containing P*_petE_* was cloned in pTA2 (T-vector; Toyobo, Osaka, Japan), and a PCR fragment containing P*_rbcL_* was cloned in pMD18-T (T-vector; Takara, Shiga, Japan). P*_petE_* was then excised and inserted downstream of P*_rbcL_* to generate P*_rbcL_*P*_petE_*. P*_rbcL_*P*_petE_*-*hetZ* and P*_rbcL_*P*_petE_*-*hetP* were generated by overlap PCR [27] and cloned into pMD-18T. P*_petE_*-*hetP* was generated by inserting the fragment containing *hetP* downstream of P*_petE_*, excised and cloned downstream of P*_rbcL_*P*_petE_*-*hetZ* to generate P*_rbcL_*P*_petE_*-*hetZ*-P*_petE_*-*hetP*. P*_rbcL_*P*_petE_*-*hetZ*, P*_rbcL_*P*_petE_*-*hetP* and P*_rbcL_*P*_petE_*-*hetZ*-P*_petE_*-*hetP* were then cloned into a shuttle vector derived from pRL25C [28], producing pHB4382, pHB4409 and pHB4551, respectively. Fragments cloned by PCR were all confirmed by sequencing. Plasmids were introduced into *Anabaena* 7120 and its derivatives by conjugation with the aid of a helper plasmid that carried methylase genes [29].

## 3. Results

### 3.1. Formation of Mch in a hetR Mutant that Overexpresses hetZ and hetP

We employed the tandem promoter P*_rbcL_*P*_petE_* to express *hetZ* and *hetP* in the *hetR* background. P*_rbcL_* is strongly expressed in vegetative cells but not expressed in heterocysts [30], and P*_petE_* is moderately expressed in both cell types [31]. Use of P*_petE_* in addition to P*_rbcL_* was to ensure that the expression of *hetZ* and *hetP* was not switched off in developing heterocysts. Because P*_rbcL_* may enhance the expression of a reporter gene by 30–40 fold [32], the tandem promoter P*_rbcL_*P*_petE_* could increase the expression to a higher level.

**Figure 1:**
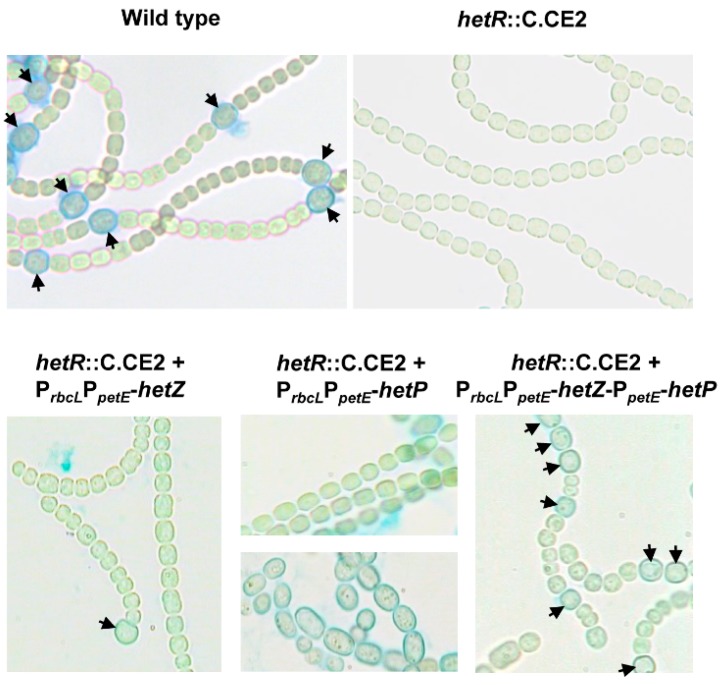
Heterocyst formation in *hetR*::C.CE2 expressing *hetZ*, *hetP* or both from P*_rbcL_*P*_petE_*. Filaments were stained by Alcian blue at 24 h after nitrogen stepdown. Most filaments of *hetR*::C.CE2 with P*_rbcL_*P*_petE_*-*hetP* showed no cell differentiation (upper panel), but a small number of filaments produced enlarged cells in strings (lower panel). Arrowheads point to mature or developing heterocysts.

The *hetR*::C.CE2 mutant carrying P*_rbcL_*P*_petE_*-*hetZ* produced heterocysts (including developing heterocysts, all but ca. 3% at the ends of filaments), and showed anoxic nitrogenase activity at 24 h after nitrogen stepdown and aerobic nitrogenase activity at 48 h (Table 2). The differentiated cells were stained by Alcian blue; they occasionally showed polar nodules, indicative of heterocysts. The frequency of differentiated cells was little changed in the second 24 h after nitrogen stepdown. The strain bearing P*_rbcL_*P*_petE_*-*hetP*, however, showed few differentiating cells in the first 24 h, but some enlarged cells in the second 24 h (Figure 1). Because it showed anaerobic nitrogenase activity (Table 2), some of those enlarged cells evidently had heterocyst-like functions. Most of such cells formed in strings in a small fraction of filaments, contained particulate inclusions, were larger, and had thicker envelopes than heterocysts.

We then tested co-expression of *hetZ* and *hetP* from the tandem promoter in the *hetR* mutant (Figure 1). In BG11 medium (with nitrate), *hetR*::C.CE2 with P*_rbcL_*P*_petE_*-*hetZ*-P*_petE_*-*hetP* produced heterocysts at a frequency of 3.0%, whereas the wild type strain produced almost no heterocysts under the same conditions. After nitrogen stepdown, the heterocyst frequency increased to 11.5% at 24 h and 37.8% at 96 h. Co-expression of *hetZ* and *hetP* resulted in higher anaerobic and aerobic nitrogenase activities than expression of *hetZ* or *hetP* alone (Table 2). However, unlike *hetR*::C.CE2 with P*_ntcA_*-*hetZ*-*hetP* in the previous report [21], this strain showed no diazotrophic growth. 

**Figure 2:**
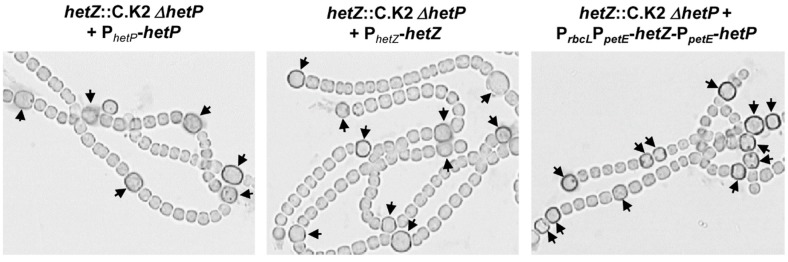
Restoration of heterocyst formation in the *hetZ*::C.K2 Δ*hetP* double mutant by expressing *hetZ*, *hetP* or both. Filaments were stained by Alcian blue at 24 h after N-stepdown. Arrowheads point to mature or developing heterocysts.

The plasmid carrying P*_rbcL_*P*_petE_*-*hetZ*-P*_petE_*-*hetP* was also introduced into the wild type and the *hetZ*::C.K2 Δ*hetP* double mutant [21]. The resulting strains also produced heterocysts in BG11 and showed increases in heterocyst frequency after nitrogen stepdown (Table 2). In the *hetZ hetP* double mutant, heterocyst formation was not restored by P*_hetR_*-*hetR* on the pDU1-based plasmid [21] but by P*_hetZ_*-*hetZ* or P*_hetP_*-*hetP* on the plasmid (Figure 2). P*_rbcL_*P*_petE_*-*hetZ*-P*_petE_*-*hetP* on the plasmid enabled the double mutant to form Mch.

### 3.2. Distribution of Heterocysts along Filaments

RGSGR has been established as an important factor in heterocyst pattern formation [16]. HetR is the only identified target for RGSGR or RGSGR-containing peptides. Now that heterocysts can be formed in a *hetR* mutant that overexpresses *hetZ* and *hetP*, it becomes feasible to test whether heterocyst formation without HetR is still susceptible to inhibition by RGSGR. Usually, heterocyst formation in the wild-type *Anabaena* 7120 can be significantly inhibited by 1 μM RGSGR and completely inhibited by 10 μM RGSGR [15]. However, RGSGR up to 50 μM showed no inhibitory effect on heterocyst formation in a *hetR* mutant that expresses *hetZ* or co-expresses *hetZ* and *hetP* (Table 2). P*_rbcL_*P*_petE_*-*hetZ*-P*_petE_*-*hetP* also enabled the wild type and the *hetZ hetP* double mutant to form heterocysts in the presence of 50 μM RGSGR (Table 2).

**Figure 3:**
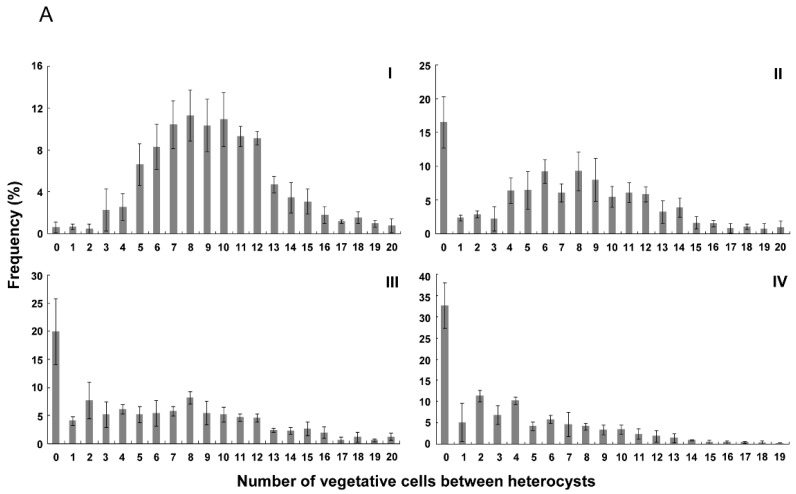

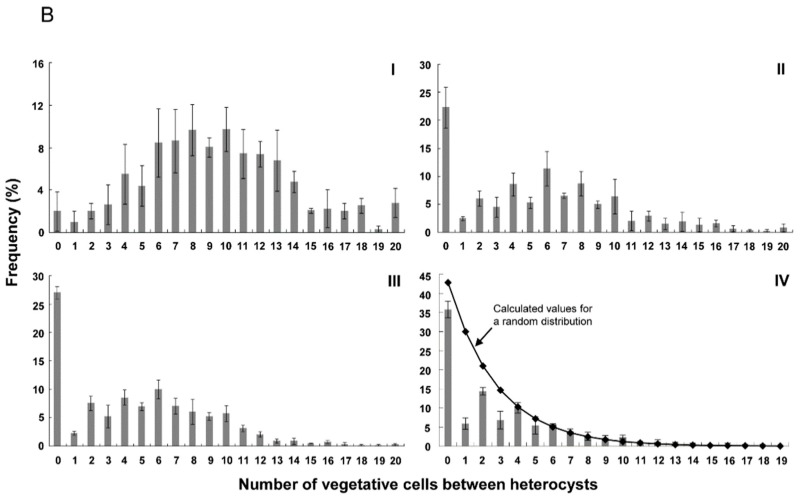
Distribution of numbers of vegetative cells between heterocysts at 24 h (**A**) and 48 h (**B**) after nitrogen stepdown. I, the wild type of *Anabaena* 7120 (WT); II, WT + P*_rbcL_*P*_petE_*-*hetZ*-P*_petE_*-*hetP*; III, *hetZ*::C.K2Δ*hetP* + P*_rbcL_*P*_petE_*-*hetZ*-P*_petE_*-*hetP*; IV, *hetR*::C.CE2 + P*_rbcL_*P*_petE_*-*hetZ*-P*_petE_*-*hetP*. Intervals longer than 19 cells are shown as 20. The calculated values for a random distribution were generated by Microsoft Excel. At 24 h after nitrogen stepdown, *hetR*::C.CE2 carrying P*_rbcL_*P*_petE_*-*hetZ*-P*_petE_*-*hetP* produced heterocysts in 69.9 ± 2.4% of filaments, and of these filaments, the heterocyst frequency was 20.5 ± 2.9%. At 48 h, it produced heterocysts in 74.7 ± 4.0% of filaments, the heterocyst frequency of these filaments was 24.0 ± 0.8%. Please note that in *hetR*::C.CE2 carrying P*_rbcL_*P*_petE_*-*hetZ*-P*_petE_*-*hetP*, filaments with heterocysts were usually shorter than those without differentiation and that heterocyst frequencies in Table 2 were calculated based on filaments with or without heterocysts.

Figure 3 shows the distribution of number of vegetative cells between heterocysts in the wild type and strains with P*_rbcL_*P*_petE_*-*hetZ*-P*_petE_*-*hetP*. As reported by Khudyakov and Golden (2004) [33], there was a bias toward even numbers of intervals, probably due to cell division after differentiation of heterocysts. In *hetR*::C.CE2 with P*_rbcL_*P*_petE_*-*hetZ*-P*_petE_*-*hetP*, more than 30% of heterocysts were formed in strings after nitrogen stepdown. At 48 h, frequencies of 0 and even number intervals in this strain were close to calculated values of a random distribution (Figure 3B-IV).

In the *hetZ hetP* double mutant with P*_rbcL_*P*_petE_*-*hetZ*-P*_petE_*-*hetP*, more than 20% of heterocysts were in strings (≥2 cells). At 24 h, intervals in the range of 2 to 12 cells were at similar frequencies (Figure 3A); at 48 h, a peak at 6 cells emerged (Figure 3B). In the wild type with P*_rbcL_*P*_petE_*-*hetZ*-P*_petE_*-*hetP*, more than 15% of heterocysts were in strings, and the frequency distribution peaked at 6-8 cells at 24 h and at 6 cells at 48 h. As a control, in the wild type of *Anabaena* 7120, frequency distribution peaked at 8-10 cells, with <2% of heterocysts in strings.

It is noteworthy that in strains with P*_rbcL_*P*_petE_*-*hetZ* or P*_rbcL_*P*_petE_*-*hetZ*-P*_petE_*-*hetP*, formation of heterocysts decreased gradually from serial subculture in BG11. Therefore, long-repeated subcultures should be avoided. In this study, heterocyst differentiation was observed with newly acquired exconjugants after two passages.

### 3.3. Heterocyst Formation in a hetF Background

**Figure 4:**
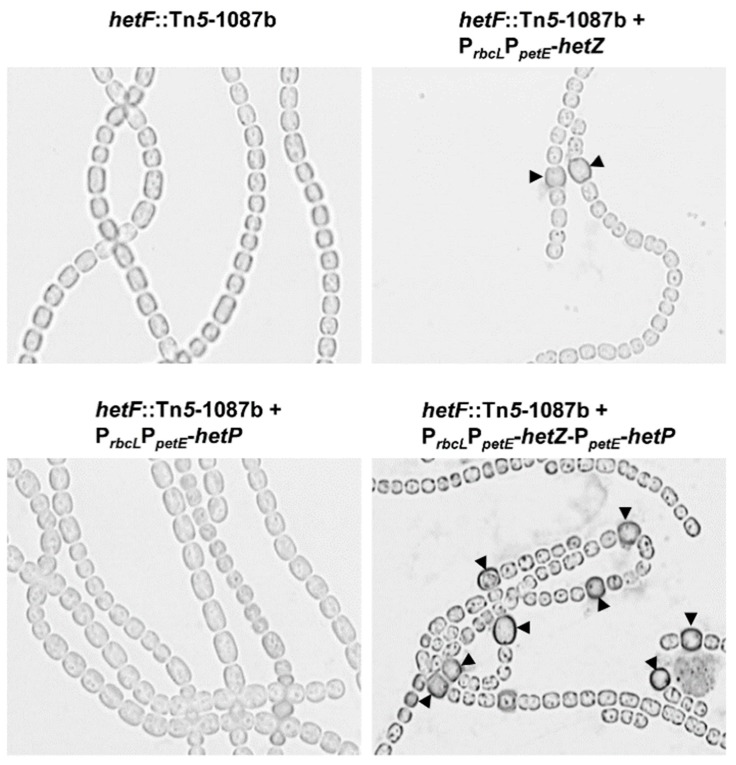
Heterocyst formation in the *hetF*::Tn*5*-1087b mutant expressing *hetZ*, *hetP* or both from P*_rbcL_*P*_petE_*. Filaments were stained by Alcian blue at 24 h after N-stepdown. Arrowheads point to mature or developing heterocysts.

In a *hetR-*minus background, expression of *hetZ* from P*_rbcL_*P*_petE_* or co-expression of *hetZ* and *hetP* from P*_ntcA_* [21] or P*_petE_* (Zhang and Xu, unpublished) restores heterocyst formation at the terminal positions of filaments. Such restoration might be due to the lack of expression of *patA* in vegetative cells [10] of a *hetR* mutant. To test this hypothesis, we introduced P*_rbcL_*P*_petE_*-*hetZ*, P*_rbcL_*P*_petE_*-*hetP* and P*_rbcL_*P*_petE_*-*hetZ*-P*_petE_*-*hetP*, respectively, into a *hetF*::Tn*5*-1087b mutant [9] to find out whether single heterocysts could be formed at intercalary positions in a *hetF* background, where *hetR* is active. Expression of *hetZ* or co-expression of *hetZ* and *hetP* restored heterocyst formation in a *hetF* mutant, whereas expression of *hetP* did not (Figure 4). In the strain expressing *hetZ*, about 47% of heterocysts were at intercalary positions (heterocyst frequency ca. 2.5% at 24 h). From 24 h to 48 h after nitrogen stepdown, the co-expressing strain showed an increase in heterocyst frequency from ca. 7.7% to ca. 21.3%, producing Mch, whereas the strains with *hetZ* or *hetP*—expressed alone—remained essentially unchanged.

## 4. Discussion

Restoration of heterocyst formation in a *hetR-*minus background has been shown in two laboratories by expression of *hetZ* and *hetP* [21] or by *hetZ* alone [23]. Even though both showed heterocyst formation without a functional *hetR*, the two reports differed in the phenotypes and genes involved. One group [23] proposed that the differences might be due to the promoters, P*_ntcA_* and P*_petE_*, used in the two studies. However, P*_petE_*had been tested in the IHB version of *Anabaena* 7120 (Zhang and Xu, unpublished) and had found results similar to those produced with P*_ntcA_*. Therefore, the differences are more likely due to divergence in genetic backgrounds of versions of *Anabaena* 7120 [22,23].

In this study, we used the P*_rbcL_*P*_petE_* tandem promoter to express the genes in the *hetR* mutant of *Anabaena* 7120 IHB and found two different patterns of heterocyst distribution, namely, with single heterocysts only at terminal positions, or with Mch at both terminal and intercalary positions. Now that heterocyst formation without HetR has been shown with moderate expression of *hetZ* and *hetP*, the use of stronger expression may further intensify the phenotype. We admit that expression of *hetZ* and *hetP* from the tandem promoter had certain side effects on cells, because the phenotypes would gradually disappear if the strains continued to be cultured and subcultured in BG11. Therefore, the observations must be performed with cultures within a suitable range of generations.

In *Anabaena* 7120 IHB, moderate co-expression of *hetZ* and *hetP* [21] or strong expression of *hetZ* (from P*_rbcL_*P*_petE_*) (Figure 1) in the *hetR* mutant led to heterocyst formation predominantly at terminal positions. Because the expression of *patA* in vegetative cells is dependent on HetR and required for heterocyst formation at the intercalary positions [10], we assumed that the *patA*-like phenotype in these strains were due to the lack of a functional HetR-*patA* system. When *hetP* was added to the expression from P*_rbcL_*P*_petE_*, heterocyst formation was so much more enhanced that the lack of a HetR-*patA* system was bypassed, and Mch were produced at intercalary and terminal positions. Consistent with the assumption, in a *hetF* mutant with P*_rbcL_*P*_petE_*-*hetZ*, where HetR-*patA* system probably remained active in vegetative cells, single heterocysts formed at both terminal and intercalary positions (Figure 4).

In parallel to the HetR-*patA* system that determines heterocyst formation at intercalary positions, the HetR-RGSGR system plays a key role in determination of heterocyst spacing. HetR is the only identified target for RGSGR-containing peptides in terms of heterocyst differentiation. Therefore, heterocyst formation without HetR may not be inhibited by RGSGR. Addition of RGSGR at a high concentration to different strains under N-deficient conditions confirmed this assumption (Table 2). Because of the lack of HetR as the sensor of RGSGR, the distribution of heterocysts in the *hetR* mutant with P*_rbcL_*P*_petE_*-*hetZ*-P*_petE_*-*hetP* (Figure 3B) resembled that of the PatS-resistant *hetRR223W* mutant described in a previous report [32]. However, in the wild type or the *hetZ hetP* double mutant that carried the same plasmid, the normal heterocyst pattern was partially maintained. To some extent, the distribution of heterocysts in the latter two strains were affected by the HetR-RGSGR system.

In summary, we have been able to generate different patterns of heterocyst distribution in *hetR* and *hetF* mutants by overexpressing *hetZ*, *hetP* or both. These results extended the previously reported findings on restoration of heterocyst formation in the *hetR* mutant [21] and provided some information that may help to understand heterocyst pattern formation, especially the phenotype of a *patA* mutant.

## Figures and Tables

**Table 1:** *Anabaena* strains used in this work.

Strains	Derivation/Relevant Characteristics ^a^	Reference or Source
*Anabaena* 7120	Wild Type (WT)	FACHB ^b^
*hetF*::Tn*5*-1087b	Em^r^, Tn*5*-1087b inserted within *hetF* 310 bp from its 3′ terminus	[9]
*hetR*::C.CE2	Cm^r^Em^r^, C.CE2 (a chloramphenicol-resistance and erythromycin-resistance cassette) inserted into the ClaI site of *hetR*	[9]
*hetR*::C.CE2 (pHB4382)	Cm^r^Em^r^Nm^r^, pHB4382 bearing P*_rbcL_*P*_petE_*-*hetZ* introduced into the *hetR*::C.CE2 mutant	This study
*hetR*::C.CE2 (pHB4409)	Cm^r^Em^r^Nm^r^Sm^r^Sp^r^, pHB4409 bearing P*_rbcL_*P*_petE_*-*hetP* introduced into the *hetR*::C.CE2 mutant	This study
*hetR*::C.CE2 (pHB4551)	Cm^r^Em^r^Nm^r^Sm^r^Sp^r^, pHB4551 bearing P*_rbcL_*P*_petE_*-*hetZ*-P*_petE_*-*hetP* introduced into the *hetR*::C.CE2 mutant	This study
*hetZ*::C.K2 *ΔhetP*	Cm^r^Em^r^Nm^r^, *hetZ hetP* double mutant	[21]
*hetZ*::C.K2 *ΔhetP* (pHB1462)	Cm^r^Em^r^Nm^r^Sm^r^Sp^r^, pHB1462 bearing P*_hetZ_*-*hetZ* [25] introduced into the *hetZ hetP* double mutant	This study
*hetZ*::C.K2 *ΔhetP* (pHB4550)	Cm^r^Em^r^Nm^r^Sm^r^Sp^r^, pHB4550 bearing P*_hetP_*-*hetP* introduced into the *hetZ hetP* double mutant	This study
*hetZ*::C.K2 *ΔhetP* (pHB4551)	Cm^r^Em^r^Nm^r^Sm^r^Sp^r^, pHB4551 bearing P*_rbcL_*P*_petE_*-*hetZ*-P*_petE_*-*hetP* introduced into the *hetZ hetP* double mutant	This study
WT (pHB4551)	Nm^r^Sm^r^Sp^r^, pHB4551 bearing P*_rbcL_*P*_petE_*-*hetZ*-P*_petE_*-*hetP* introduced into *Anabaena* 7120	This study

^a^ Cm, chloramphenicol; Em, erythromycin; Nm, neomycin; Sm, streptomycin; Sp, spectinomycin; stated otherwise, the P*_rbcL_*, P*_hetZ_*, and P*_hetP_*templates for PCR reactions were *Anabaena* 7120 genomic DNA; ^b^ FACHB, Freshwater Algal Culture Collection of the Institute of Hydrobiology, Chinese Academy of Sciences.

**Table 2:** Nitrogenase activities and heterocyst frequencies of *Anabaena* strains after nitrogen stepdown.

Strains	Hours	Nitrogenase Activity (Mole C_2_H_4_ mg Chl*a*^−1^ h^−1^) ^a^		Heterocyst Frequency (%) ^b^		Diazotrophic Growth
		Anoxic	Aerobic	−RGSGR	+RGSGR ^c^	
WT ^d^	24	3.65 ± 0.62	2.83 ± 0.11	9.4 ± 0.4	0	Good
	48	6.74 ± 0.63	3.50 ± 0.57	10.6 ± 0.7	Not tested	
WT + P*_rbcL_*P*_petE_*-*hetZ*-P*_petE_*-*hetP* ^e1^	24	Not measured	Not measured	11.9 ± 1.2	11.4 ± 1.3	Moderate
	48	Not measured	Not measured	15.8 ± 2.8	Not tested	
*hetR*::C.CE2	24	0	0	0	Not tested	No
	48	0	0	0	Not tested	
*hetR*::C.CE2 + P*_rbcL_*P*_petE_*-*hetZ*	24	1.04 ± 0.14	0	2.9 ± 0.9	3.6 ± 0.4	No
	48	1.20 ± 0.46	0.81 ± 0.13	3.5 ± 0.9	Not tested	
*hetR*::C.CE2 + P*_rbcL_*P*_petE_*-*hetP*	24	0.17 ± 0.10	0	Not counted ^f^	Not tested	No
	48	1.57 ± 0.26	0	Not counted	Not tested	
*hetR*::C.CE2 + P*_rbcL_*P*_petE_*-*hetZ*-P*_petE_*-*hetP*^e2^	24	4.00 ± 1.86	0.90 ± 0.07	11.5 ± 2.0	12.9 ± 0.9	No
	48	4.12 ± 0.27	1.52 ± 0.78	17.5 ± 2.0	Not tested	
*hetZ*::C.K2 *ΔhetP*	24	0	0	0	Not tested	No
	48	0	0	0	Not tested	
*hetZ*::C.K2 *ΔhetP* + P*_rbcL_*P*_petE_*-*hetZ*-P*_petE_*-*hetP* ^e3^	24	Not measured	Not measured	14.3 ± 1.2	15.7 ± 0.7	Moderate
	48	Not measured	Not measured	17.6 ± 0.4	Not tested	

^a^ Nitrogenase activity was evaluated based on acetylene reduction over 6 h. ^b^ Mature and developing heterocysts were both included. ^c^ RGSGR was added to BG11_0_ at a final concentration of 50 M. ^d^ Nitrogenase activity and heterocyst frequency of the wild type varied between different batches of experiments under the described conditions. For example, at 24 h, the nitrogenase activity under aerobic conditions may change in a range from 2.8 to 9.1 μmole C_2_H_4_ mg Chl*a*^−1^ h^−1^, and heterocyst frequency may change from 8.7 to 10.8%. Data presented are from one batch of experiments. ^e1~e3^ These strains produced heterocysts in BG11 (with nitrate) at 7.4 ± 0.9%, 3.0 ± 0.5% and 6.1 ± 1.0% respectively, while all other strains produced heterocysts at 0~0.4% under the same conditions. ^f^ It was difficult to distinguish N_2_-fixing cells from those enlarged cells in this strain.

## Supplementary Materials

The following are available online at http://www.mdpi.com/2075-1729/8/4/60/s1, Table S1: A list of plasmids and primers.
