# Supplementary material for: Manipulation of Pattern of Cell Differentiation in a hetR Mutant of Anabaena sp. PCC 7120 by Overexpressing hetZ Alone or with hetP
Source: Life (Basel). 2018 Nov 30;8(4):60. doi: 10.3390/life8040060 (PMC6316738; doi:10.3390/life8040060)
Supplement: Supplementary file 1 [file life-08-00060-s001.pdf]

TABLE S1 A list of plasmids and primers

Plasmids and primers	Derivation, relevant characteristics[a] or sequences
**Plasmids**	**Derivation, relevant characteristics**
pHB1462	Sm^rSp^r, P_{hetZ}-*hetZ* cloned upstream of *gfp* in a pDU1-based shuttle vector (Zhang et al., 2007).
pHB3729	$Nm^rSm^rSp^r$, the Ω cassette excised with DraI from pRL57, blunted with T4 DNA polymerase, cloned into BamHI-cut and T4 DNA polymerase-blunted pRL25C, a pDU1-based shuttle vector (Wolk et al., 1988)
pHB4198	Ap^r, the PCR fragment (*Anabaena* sp. chromosomal bp 277818-278291) containing P_{petE} amplified using primers PpetE-F and PpetE-R, cut with PstI/XbaI, cloned into PstI/XbaI-cut pTA2
pHB4219a	Ap^r, the PCR fragment (*Anabaena* sp. chromosomal bp 3432367-3432843) containing the *hetP* coding region amplified using primers 2818-F and 2818-R, cloned into pMD18-T
pHB4219b	Ap^r, *hetP* coding region excised with NdeI/EcoRI from pHB4219a, cloned into NdeI/EcoRI-cut pHB4198
pHB4220	Ap^r, the PCR fragment (*Anabaena* sp. chromosomal bp 1784801-1785483) containing P_{rbcL} amplified using primers PrbcL-1 and PrbcL-2, cloned into pMD18-T
pHB4221	Ap^r, P_{petE} excised with BamHI/EcoRI from pHB4198, cloned into BamHI/EcoRI-cut pHB4220, generating $P_{rbcL}P_{petE}$
pHB4331	Ap^r, $P_{rbcL}P_{petE}$-*hetP*, generated by overlap PCR (Horton et al., 1989) using two primer pairs PrbcL-1/2818-PpetE-1 and PpetE-2818-2/2818-R, pHB4221 as the template for $P_{rbcL}P_{petE}$ and chromosomal DNA as the template for *hetP*, cloned into pMD18-T
pHB4332	Ap^r, $P_{rbcL}P_{petE}$-*hetZ*, generated by overlap PCR using two primer pairs PrbcL-1/0099-PpetE-1 and PpetE-0099-2/0099-R, pHB4221 as the template for $P_{rbcL}P_{petE}$ and chromosomal DNA as the template

	for *hetZ*, cloned into pMD18-T
pHB4343	Ap^{r}Cm^{r}Em^r, C.CE2 excised with SalI from pRL598, inserted into SalI-cut pHB4332, upstream of P$_{rbcL}$P$_{petE}$-*hetZ*
pHB4382	Cm^{r}Em^{r}Km^r, C.CE2-P$_{rbcL}$P$_{petE}$-*hetZ* excised with KpnI/SphI from pHB4343, blunted with T4 DNA polymerase, cloned into BamHI-cut and T4 DNA polymerase-blunted pRL25C
pHB4409	Km^{r}Sm^{r}Sp^r, P$_{rbcL}$P$_{petE}$-*hetP* excised with PvuII from pHB4331, cloned into EcoRI-cut and T4 DNA polymerase blunted pHB3729
pHB4539	Ap^r, P$_{petE}$-*hetP* excised with BamHI/EcoRI from pHB4219b, blunted with T4 DNA polymerase, cloned into SmaI-cut pHB4332, oriented as P$_{rbcL}$P$_{petE}$-*hetZ*, generating P$_{rbcL}$P$_{petE}$-*hetZ*-P$_{petE}$-*hetP*
pHB4550	Km^{r}Sm^{r}Sp^r, P$_{hetP}$-*hetP* carried on a pDU1-based shuttle vector (Zhang et al., 2018)
pHB4551	Km^{r}Sm^{r}Sp^r, P$_{rbcL}$P$_{petE}$-*hetZ*-P$_{petE}$-*hetP* excised with KpnI/SphI from pHB4539, blunted with T4 DNA polymerase, cloned into EcoRI-cut and T4 DNA polymerase blunted pHB3729
pMD18-T	Ap^r, T-vector (TaKaRa)
pRL25C	Km^r(Nm^r), pDU1-based shuttle vector (Wolk et al., 1988)
pRL598	Cm^{r}Em^r, cloning vector with the C.CE2 cassette (Black and Wolk, 1994)
pTA2	Ap^r, T-vector (Toyobo)

**Primers**	**Sequences (5′→3′)**
0099-PpetE-1	taggagaacgcatatgaactcagccgcaac
0099-R	aaagaattcctgagtcactattcatgag
2818-PpetE-1	taggagaacgcatatgaaccaaaacactacaggc
2818-F	aaacatatgaaccaaaacactacaggcataac
2818-R	ggcgaattctcaattatgaataaaatctaggtctgac
PpetE-0099-2	gttgcggctgagttcatatgcgttctccta

PpetE-2818-2	gcctgtagtgttttggttcatatgcgttctccta
PpetE-F	ctttctagaggatcctaaagcctgtgaa (Sakr et al., 2006)
PpetE-R	cttctgcagcatatgcgttctcctaacctgtagttt (Sakr et al., 2006)
PrbcL-1	aagatatccgccaagaacctacccag
PrbcL-2	acagatctccacgtaatttgtaacttg

[a] Ap, ampicillin; Cm, chloramphenicol; Em, erythromycin; Km, kanamycin; Nm, neomycin; Sm, streptomycin; Sp, spectinomycin.

REFERENCES

Black TA, Wolk CP. 1994. Analysis of a Het^{-} mutation in *Anabaena* sp. strain PCC 7120 implicates a secondary metabolite in the regulation of heterocyst spacing. J. Bacteriol. **176:** 2282-2292.

Horton R, Hunt HD, Ho SN, Pullen JK, Pease LR. 1989. Engineering hybrid genes without the use of restriction enzymes: gene splicing by overlap extension. Gene **77:** 61-68.

Sakr S, Jeanjean R, Zhang C-C, Arcondeguy T. 2006. Inhibition of cell division suppresses heterocyst development in *Anabaena* sp. strain PCC 7120. J. Bacteriol. **188:** 1396-1404.

Wolk CP, Cai Y, Cardemil L, Flores E, Hohn B, Murry M, Schmetterer G, Schrautemeier B, Wilson R. 1988. Isolation and complementation of mutants of *Anabaena* sp. strain PCC 7120 unable to grow aerobically on dinitrogen. J. Bacteriol. **170:** 1239-1244.

Zhang W, Du Y, Khudyakov I, Fan Q, Gao H, Ning D, Wolk CP, Xu X. 2007. A gene cluster that regulates both heterocyst differentiation and pattern formation in *Anabaena* sp. strain PCC 7120. Mol. Microbiol. **66:** 1429-1443.

Zhang H, Wang S, Wang Y, Xu X. 2018. Functional overlap of *hetP* and *hetZ* in regulation of heterocyst differentiation in *Anabaena* sp. strain PCC 7120. J. Bacteriol. **200:** e00707-17.
